# Skeletal Muscle Atrophy Was Alleviated by Salidroside Through Suppressing Oxidative Stress and Inflammation During Denervation

**DOI:** 10.3389/fphar.2019.00997

**Published:** 2019-09-10

**Authors:** Ziwei Huang, Qingqing Fang, Wenjing Ma, Qiuyu Zhang, Jiaying Qiu, Xiaosong Gu, Huilin Yang, Hualin Sun

**Affiliations:** ^1^Department of Orthopedics, Orthopedic Institute, the First Affiliated Hospital, Soochow University, Suzhou, China; ^2^Key Laboratory of Neuroregeneration of Jiangsu and Ministry of Education, Jiangsu Clinical Medicine Center of Tissue Engineering and Nerve Injury Repair, Co-Innovation Center of Neuroregeneration, Nantong University, Nantong, Jiangsu China

**Keywords:** salidroside, nerve injury, muscle atrophy, oxidative stress, inflammation

## Abstract

Skeletal muscle atrophy is a common and debilitating condition that lacks an effective therapy. Oxidative stress and inflammation are two main molecular mechanisms involved in muscle atrophy. In the current study, we want to explore whether and how salidroside, with antioxidant and anti-inflammatory properties, protects against skeletal muscle atrophy induced by denervation. First, oxidative stress and inflammatory response were examined during myotube atrophy induced by nutrition deprivation. The results demonstrated that oxidative stress and inflammatory response were induced in cultured myotubes suffered from nutrition deprivation, and salidroside not only inhibited oxidative stress and inflammatory response but also attenuated nutrition deprivation-induced myotube atrophy, as evidenced by an increased myotube diameter. The antioxidant, anti-inflammatory, and antiatrophic properties of salidroside in cultured myotubes were confirmed in denervated mouse models. The mice treated with salidroside showed less oxidative stress and less inflammatory cytokines, as well as higher skeletal muscle wet weight ratio and larger average cross sectional areas of myofibers compared with those treated with saline only during denervation-induced skeletal muscle atrophy. Moreover, salidroside treatment of denervated mice resulted in an inhibition of the activation of mitophagy in skeletal muscle. Furthermore, salidroside reduced the expression of atrophic genes, including MuRF1 and MAFbx, autophagy genes, including PINK1, BNIP3, LC3B, ATG7, and Beclin1, and transcription factor forkhead box O3 A (Foxo3A), and improved the expression of myosin heavy chain and transcriptional factor phosphorylated Foxo3A. Taken together, these results suggested that salidroside alleviated denervation-induced muscle atrophy by suppressing oxidative stress and inflammation.

## Introduction

Skeletal muscle is the largest tissue in the body, and loss of its function results in debilitating musculoskeletal disorders, impairing quality of life, increasing morbidity and mortality, and bringing the economic and social burden for families and communities (Chal and Pourquie, 2017; Qiu et al., 2018). However, the treatment of skeletal muscle wasting remains an unresolved challenge to this day (Dutt et al., 2015). Therefore, elucidating its molecular basis and developing treatment strategies to prevent or even reverse the atrophic process has become the focus of current research.

Oxidative stress and inflammation are two main molecular mechanisms involved in muscle atrophy (van Helvoort et al., 2007; Kim et al., 2015; Anderson et al., 2017). Growing evidences suggested that increased reactive oxygen species (ROS) production in skeletal muscles significantly induced mitochondrial dysfunction, activated forkhead box class O (FoxO) transcription factors, and contributed to inactivity-induced muscle atrophy (McClung et al., 2007; Powers et al., 2012; Matsui, 2017). Our previous study also showed that ROS played a crucial role in denervation-induced muscle atrophy (Qiu et al., 2018). Inflammation is an important pathogenic factor, which contributes to the dysfunction of skeletal muscle. It impairs muscle homeostasis and myogenesis, also activates the family of FoxO transcription factors, and contributes to skeletal muscle atrophy (Costamagna et al., 2015; Milan et al., 2015). In summary, we speculate that elevated ROS and activated inflammation may trigger downstream proteolysis pathways in denervated skeletal muscles, likely through activating FoxO. Therefore, oxidative stress and inflammation might be a potential therapeutic target against denervation-induced skeletal muscle atrophy.

The activation of FoxO was essential for the muscle atrophy induced by denervation or fasting, and activated FoxO3 caused coordinately activating ubiquitin–proteasome system (UPS) and autophagy–lysosomal system (ALS), especially lysosomal proteolysis, in denervation- or fasting-induced muscles atrophy (Zhao et al., 2007; Zhao et al., 2008). Moreover, FoxO3 activation stimulated autophagy through a transcription-dependent mechanism and upregulated the transcription of autophagy-related genes (Zhao et al., 2008). FoxO knockout could reverse the muscle loss and markers of increased ubiquitin–proteasome degradation and autophagy alterations induced by streptozotocin-diabetes mice (O’Neill et al., 2019). Thus, it can be seen that the activated FoxO was essential for the activation of UPS and ALS in muscle atrophy.

Skeletal muscle atrophy occurs when the rates of protein degradation exceed the rates of protein synthesis, and excessive protein degradation is the main cause for muscle dysfunction (Theilen et al., 2017; Cai et al., 2018). The UPS and ALS are the two major proteolytic pathways involved in muscle atrophy (Sun et al., 2014; Qiu et al., 2018). Denervation could activate the proteolytic pathways UPS and ALS through inducing the expression of muscle-specific E3 ubiquitin ligases and autophagy proteins in skeletal muscle (Paul et al., 2010). Muscle-specific ubiquitin ligase muscle atrophy F-Box (MAFbx)/atrogin-1 and muscle ring-finger-1 (MuRF-1) are key enzymes of UPS, while PINK1, BNIP3, LC3B, ATG7, and Beclin1 are key effectors of ALS (Giron et al., 2015; Ninfali et al., 2018; Qiu et al., 2018). In summary, skeletal muscle atrophy was accompanied by the activation of proteolytic pathways UPS and ALS, as evidenced by elevated levels of key enzymes of UPS and key effectors of ALS.

Salidroside, a biologically active ingredient of *Rhodiola rosea*, becomes an attractive bio-agent due to its multifunction, including anti-inflammatory, antioxidative, and antiapoptotic properties (Mao et al., 2015). Previous studies have demonstrated that salidroside protected against 1-methyl-4-phenylpyridinium-induced Parkinson’s disease in PC12 cells through inhibiting oxidative stress and inflammation (Zhou et al., 2019), as well as promoted random skin flap survival by alleviating inflammation and oxidative stress in mice (Deheng et al., 2016). Zhang et al. indicated that salidroside might have a protective effect against Alzheimer’s disease *via* modulating oxidative stress and inflammatory mediators (Zhang et al., 2013). However, it is not clear whether salidroside could protect against denervation-induced skeletal muscle atrophy through alleviating oxidative stress and inflammation. Hence, we aimed to test whether salidroside attenuates denervation-induced skeletal muscle atrophy, and if so, to clarify whether salidroside exerts its positive effect through modulating oxidative stress and inflammation.

## Materials and Methods

### Animal Treatment

This study was carried out in accordance with the recommendations of the Institutional Animal Care and Use Committee of Nantong University (No. 20170305-003). The protocol was approved by the Institutional Animal Care and Use Committee of Nantong University. Animals in experimental groups were subjected to unilateral sciatic nerve transection under anesthesia as described previously (Qiu et al., 2018), followed by daily intraperitoneal injection of saline (100 μL; NS group), salidroside (5, 10, and 20 mg/kg; Sigma-Aldrich) in saline (Sal L, Sal M, Sal H group), or ROS scavenger N-acetyl-cysteine (NAC) (20 mg/kg; Sigma-Aldrich) in saline (NAC group), respectively. Animals in normal control group received sham-operation and then injected with the same amount of saline daily (Ctrl group). After 14 days, mice were anesthetized, and tissues were removed, weighed, and snap-frozen in liquid nitrogen before storing at −80°C.

### Cell Culture and Treatments

Briefly, C2C12 myoblast cells were maintained in Dulbecco’s modified Eagle’s medium (DMEM) supplemented with 10% fetal bovine serum (FBS; (Gibco Company), 100 U/mL of penicillin, and 100 μg/mL of streptomycin in a humidified atmosphere of 5% CO_2_ at 37°C. To induce differentiation, C2C12 myoblast cells differentiated into myotubes in the presence of 2% horse serum (American Type Culture Collection, Manassas, VA, USA) for 7 days, and the differentiated media was changed every 2 days until the end of the experiment (Sun et al., 2014). Then the differentiated C2C12 myotubes were incubated for 12 h with or without the presence of salidroside (Sal L: 40 μM, Sal M: 80 μM, Sal H: 160 μM) or NAC (5 mM) dissolved in amino acid-free and serum-free Hank’s balanced salt solution (HBSS; Gibco Company) as described previously for 12 h (Qiu et al., 2018). After treatment, the C2C12 myotubes were examined by morphometric or biochemical assays.

### qRT-PCR

Total RNA was extracted using the RNeasy kit (Qiagen, Valencia, CA, USA), cDNA was synthesized using the first-strand cDNA synthesis kit with oligo dT primers (Invitrogen, Carlsbad, CA, USA), and RT-PCR was performed using the iTaq Fast SYBR Green Supermix (Bio-Rad, Hercules, CA, USA) exactly following the manufacturer’s instructions. Quantitative data of mRNA expressions were acquired and analyzed using an Applied Biosystems 7500 real-time PCR system (Applied Biosystems, Foster City, CA, USA). The RT-PCR conditions were as follows: 42°C for 20 min and then 40 cycles at 95°C for 5 min, 94°C for 20 s, and 72°C for 42 s. The melting curve was run at 65°C to 95°C. The primers were as follows: mouseNrf2F: GTTGCCCACATTCCCAAACA, R: CTGATGAGGGGCAGTGAAGA; mouseNox2F: AGTGCGTGTTGCTCGACAA, R: GCGGTGTGCAGTGCTATCAT; mouse Nox4F: CCTCCTGGCTGCATTAGTCT, R: CAGGTCTGTGGG AAATGAGC; mouseNQO1F: AGGATGGGAGGTACTCGAA TC, R: TGCTAGAGATGACTCGGAAGG; mouseHO-1F: AGG TACACATCCAAGCCGAGA, R: CATCACCAGCTTAAAGCC TTCT; mouse IL-1βF: GAAATGCCACCTTTTGACAGTG,R: TGGATGCTCTCATCAGGACAG; mouseIL-6F: CTGCAAGA GACTTCCATCCAG, R:AGTGGTATAGACAGGTCTGTTGG, mouseTNF-αF: CAGGCGGTGCCTATGTCTC, R: CGATCAC CCCGAAGTTCAGTAG; mouseGAPDHF: CATGGCCTTCC GTGTTCCTA, R: GCGGCACGTCAGATCCA. The relative mRNA expression was measured through the 2^−ΔΔCt^ method (Livak and Schmittgen, 2001).

### Western Blot Analysis

Western blot analysis was performed as described previously (Qiu et al., 2018). The proteins from skeletal muscles were extracted with radio immunoprecipitation assay (RIPA) buffer and proteins were then separated by electrophoresis on a sodium dodecyl sulfate-polyacrylamide (SDS-PAGE) gel. After, the proteins were transferred to polyvinylidene fluoride membranes (Millipore Corp, Billerica, MA, USA), which were blocked with 5% nonfat dry milk in Tris-buffer saline (TBS) followed by incubation with primary antibodies: mouse anti-MHC polyclonal antibody (T421/S424) (R&D Systems, Minneapolis, MN, USA), rabbit anti-NOX4 polyclonal antibody (Invitrogen, Rockford, IL, USA), mouse anti-BNIP3 and goat anti-forkhead box O3 A (anti-FOXO3A) antibodies (Abcam, Cambridge, UK), rabbit anti-MURF1, MAFbx (Fbx32), LC3B, PINK1, ATG7, Beclin1, NOX2, Nrf2, NQO1, FOXO3A (phospho S253), and beta tubulin antibodies (Abcam, Cambridge, UK) at 4°C overnight. Then, the membranes were probed with the horseradish peroxidase-conjugated secondary antibodies for 2 h. Immunoactive bands were visualized by enhanced chemiluminescence (Thermo Scientific, Park Ellisville, MO, USA), and the intensity values were obtained for further normalization against loading control.

### Immunofluorescence and Quantification of Myotubes

The myotube diameter was detected using MHC staining. Briefly, C2C12 cells were grown and differentiated into myotubes. Then the C2C12 myotubes were fixed in 4% paraformaldehyde, permeabilized in 0.1% saponin, blocked in 1% BSA, and then incubated overnight at 4°C with mouse anti-MHC antibody (1:200; R&D Systems). Myotubes were then rinsed with phosphate-buffered saline (PBS) and incubated for 30 min with 1:100 affinity-purified Alexa Fluor dye-conjugated goat anti-mouse antibody (Life Technologies, Carlsbad, CA, USA) at room temperature. Immunostained myotubes were visualized under a fluorescence microscope (Zeiss, Germany). To estimate myotube size, myotubes were defined as all multinucleated cells positive for the MHC stain and containing at least three nuclei. The diameter of at least 100 myotubes per condition was measured using ImageJ software (NIH, Bethesda, MD, USA), and the average diameter per myotube was calculated as the mean of three measurements taken along the length of the myotube (Abrigo et al., 2016).

### Muscle Fiber CSA Size

The fiber CSA of TA muscles was detected using laminin staining. Briefly, mouseTA muscles were fixed in 4% paraformaldehyde at room temperature. Subsequently, TA muscles were flash frozen in embedding medium and sectioned on cryostat with 10-μm thickness. The cryosections were placed on glass slides. After blocking and washing, the slides were incubated for 12 h at 4°C with anti-laminin antibody (1:200; Abcam, Cambridge, UK). Sections were subsequently incubated with the Alexa Fluor secondary antibody (1:400; Invitrogen Antibodies, Waltham, MA, USA) for 30 min at room temperature. Then, the slides were imaged by fluorescence microscopy (Zeiss, Germany), and the CSA of myofibers were determined through a blinded analysis with the ImageJ software (NIH, Bethesda, MD, USA) of five randomly captured muscle images from each experimental condition.

### Enzyme-Linked Immunosorbent Assay

ELISA plates (Beyotime, Haimen, P. R. China) were washed with wash buffer five times and incubated with 100 μL of muscle lysates from the TA muscles or 100 μL of C2C12 myotube lysates for 2 h at 37°C. Following, ELISA plates were washed and incubated with biotinylated polyclonal anti-interleukin 1 beta (anti-IL1β), anti-IL6, or anti-TNF-α antibodies for 1 h at 37°C. Subsequently, ELISA plates were washed and incubated with HRP-streptavidin for 20 min at 37°C in the dark. Finally, enzyme activity was measured using TMB at 450 nm.

### Transmission Electron Microscopy (TEM) Analysis

To observe the changes in the mitochondria, the TA muscle was analyzed through TEM analysis. The detailed procedures of TEM for muscle were previously reported (Wang et al., 2018). Briefly, 1-mm^3^-sized muscle was fixed in 2.5% glutaraldehyde followed by post fixation in 1% osmium tetroxide. Muscle sections were analyzed by TEM (HT7700, Hitachi, Tokyo, Japan). A total of 20 fields per mouse performed in three mice per condition were analyzed.

### Determination of ROS

The ROS level in C2C12 myotubes or in TA muscles was measured using dichlorodihydrofluorescein (DCFH-DA) or dihydroethidium (DHE) staining (Sigma-Aldrich), respectively (Qiu et al., 2018). Briefly, C2C12 myotubes were washed with PBS and fresh DMEM without phenol red, and incubated with 10 μM of DCFH-DA for 20 min at room temperature in the dark. Then the ROS production was measured by DCF fluorescence at an excitation wavelength of 488 nm and an emission wavelength of 519 nm. To perform DHE staining on TA muscles, animals were perfused with DHE (10 μM). Then, TA muscles were obtained, sectioned, and observed under fluorescence microscopy (Zeiss, Germany). The individual value of fluorescence intensity was normalized against that measured in untreated controls.

### Statistical Analysis

All data were analyzed using one-way ANOVA, followed by the Tukey’s multiple comparisons test to detect differences between groups. All statistical analyses were conducted with GraphPad Prism software (version 7.0; San Diego, CA, USA). A value of *p* < 0.05 was considered statistically significant.

## Results

### Salidroside Alleviates Myotube Atrophy Induced by Nutrition Deprivation

**Figure 1:**
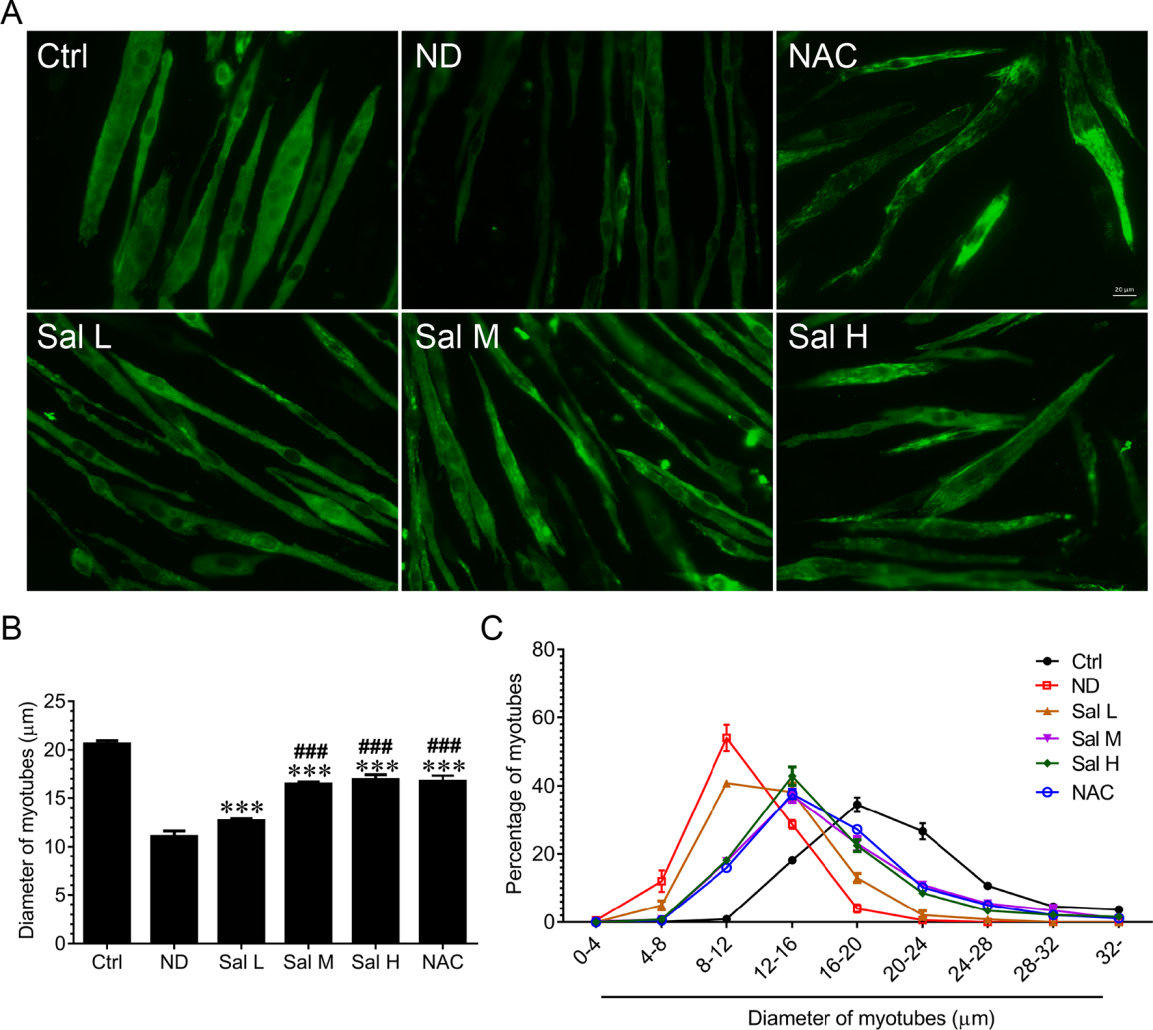
Salidroside alleviated nutrition deprivation-induced myotube atrophy. **(A)** Representative images of myosin heavy chain (MHC)-stained myotubes at 12 h following treatment with Hank’s balanced salt solution (HBSS) with or without the presence of salidroside (Sal L: 40 μM, Sal M: 80 μM, Sal H: 160 μM) or NAC (5 mM). Green indicates MHC staining. Scale bar, 20 μm. **(B)** Quantification of myotube size. At least 100 myotubes were analyzed for each group. Mean ± SD of diameter of myotubes. **(C)** Frequency distribution of diameter of myotubes. Data are expressed as mean ± SD. ***p < 0.001 versus the normal control (Ctrl) group. ^###^p < 0.001 versus the (nutrition deprivation) ND group.

To evaluate whether salidroside could protect against myotube atrophy induced by nutrition deprivation, C2C12 myotubes were incubated in HBSS for 12 h with or without the presence of salidroside (Sal L: 40 μM, Sal M: 80 μM, Sal H: 160 μM) or NAC (5 mM), after which the C2C12 myotubes were stained with MHC. The results showed that nutrition deprivation markedly induced the C2C12 myotube atrophy, as evidenced by the decreased myotube diameter. Interestingly, salidroside could alleviate myotube atrophy induced by nutrition deprivation, which was confirmed by the increased myotube diameter, and the frequency distribution of the myotube diameter shifted toward bigger sizes. Moreover, the administration of salidroside at 40 μM and 160 μM showed dose dependence, and high doses of salidroside (160 μM) showed better performance in alleviating myotube atrophy. Intriguingly, treatment with NAC and ROS scavenger obtained similar results with high doses of salidroside in nutrition deprivation-induced myotube atrophy (**Figure 1**).

### Salidroside Inhibits Oxidative Stress and Inflammaton in Nutrition Deprivation-Induced Myotube Atrophy

**Figure 2:**
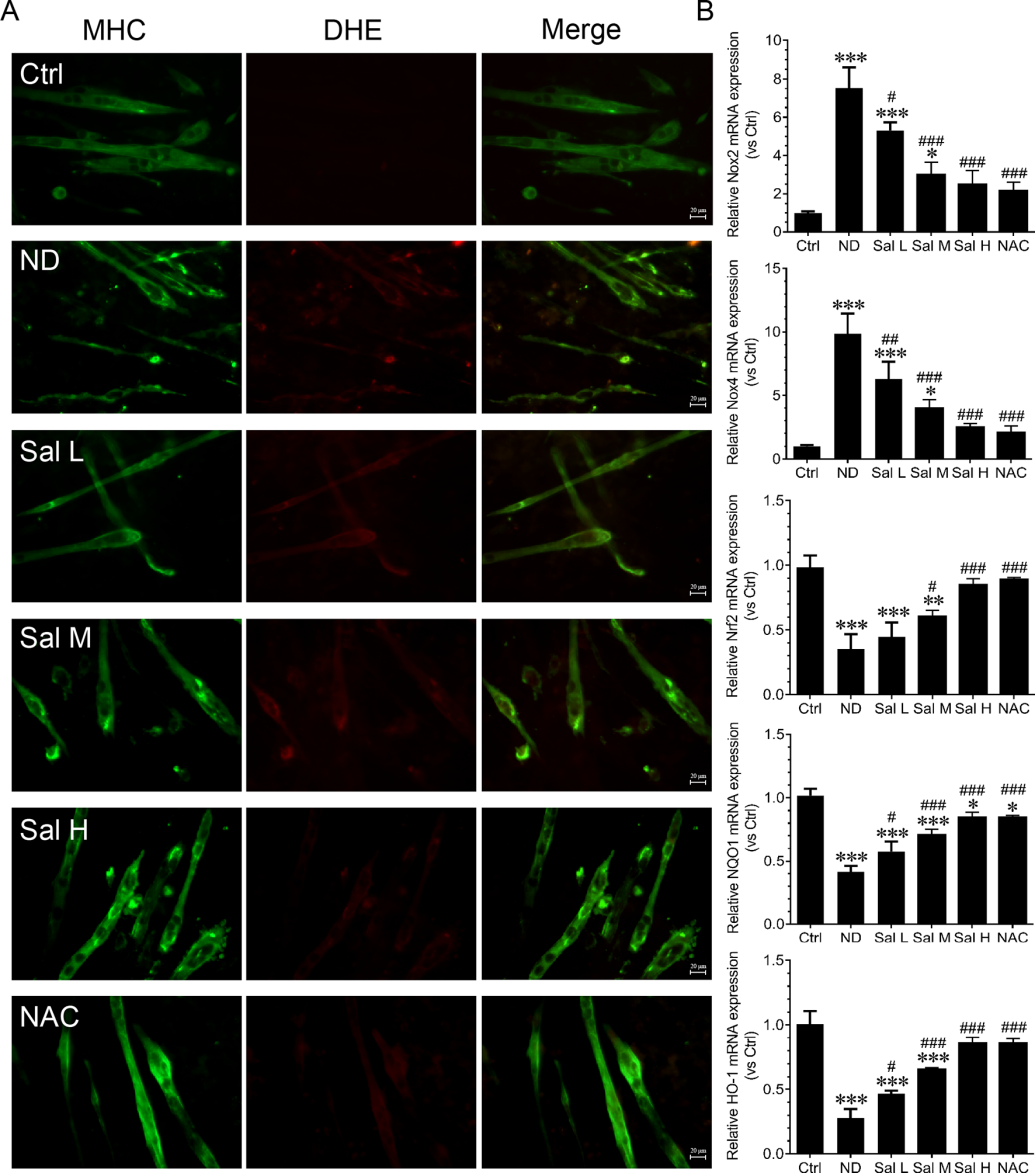
Salidroside inhibited oxidative stress in cultured myotubes suffered from nutrition deprivation. **(A)** Representative images of dihydroethidium (DHE)-stained myotubes at 12 h following treatment with HBSS with or without the presence of salidroside (Sal L: 40 μM, Sal M: 80 μM, Sal H: 160 μM) or NAC (5 mM).Green and red indicate MHC staining and DHE staining, respectively. Scale bar, 20 μm. **(B)** The qRT-PCR analyses of the expression of oxidative stress-related genes Nox2, Nox4, Nrf2, NQO1, and HO-1 mRNA in myotubes. Data are expressed as mean ± SD. *p < 0.05, **p < 0.01, and ***p < 0.001 versus the normal control (Ctrl) group. ^#^p < 0.05, ^##^p < 0.01, and ^###^p < 0.001 versus the (nutrition deprivation) ND group.

Oxidative stress and inflammation are two main molecular mechanisms involved in muscle atrophy (van Helvoort et al., 2007; Kim et al., 2015; Anderson et al., 2017). To evaluate whether salidroside attenuates myotube atrophy induced by nutrition deprivation through inhibiting oxidative stress and inflammation, we examined ROS levels and inflammatory cytokines. The current study indicated that ROS were markedly induced in the C2C12 myotubes treated with HBSS, as evidenced by the increased DHE fluorescence. Salidroside inhibited the production of ROS in C2C12 myotubes treated with HBSS, which was confirmed by the decreased DHE fluorescence. Moreover, the high doses of salidroside showed better performance in cleaning up ROS, which was similar to the response from NAC in nutrition deprivation-induced myotube atrophy (**Figure 2**). At the same time, Nox2, Nox4, Nrf2, NQO1, and HO-1 mRNA were determined. Our results suggested that Nox2 and Nox4 mRNA were markedly induced, and Nrf2, NQO1, and HO-1 mRNA were markedly suppressed in the C2C12 myotubes treated with HBSS. Salidroside could reverse the response from nutrition deprivation (**Figure 2**).

**Figure 3:**
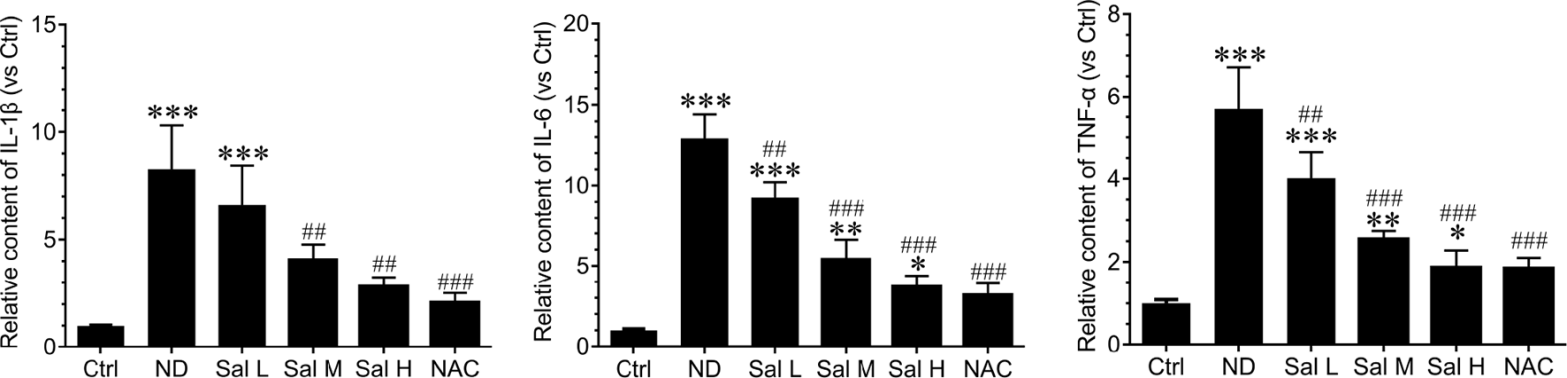
Salidroside inhibited inflammatory cytokines in cultured myotubes suffered from nutrition deprivation. The content of inflammatory cytokines, including interleukin 1 beta (IL-1β), interleukin-6 (IL-6), and tumor necrosis factor alpha (TNF-α), were determined by enzyme-linked immunosorbent assay (ELISA) in the myotubes at 12 h following treatment with HBSS with or without the presence of salidroside (Sal L: 40 μM, Sal M: 80 μM, Sal H: 160 μM), or N-acetyl-cysteine (NAC) (5 mM). Data are expressed as mean ± SD.*p < 0.05, **p < 0.01, and ***p < 0.001 versus the normal control (Ctrl) group. ^##^p < 0.01, and ^###^p < 0.001 versus the (nutrition deprivation) ND group.

Furthermore, our data also demonstrated that inflammation was significantly induced during nutrition deprivation-induced myotube atrophy, as evidenced by the increased levels of inflammatory cytokines, such as IL-1β, IL-6, and TNF-α. Salidroside could decrease the expression of inflammatory cytokines, and salidroside showed dose dependence, and high doses of salidroside showed better performance in inhibiting inflammation (**Figure 3**). Interestingly, the effects of salidroside inhibiting inflammation were similar to the response from NAC, which also suggested that oxidative stress may be upstream of the inflammation. These data suggest that salidroside-attenuated myotube atrophy is mediated, at least in part, through inhibiting oxidative stress and inflammation.

### Salidroside Attenuates Denervation-Induced Skeletal Muscle Atrophy

**Figure 4:**
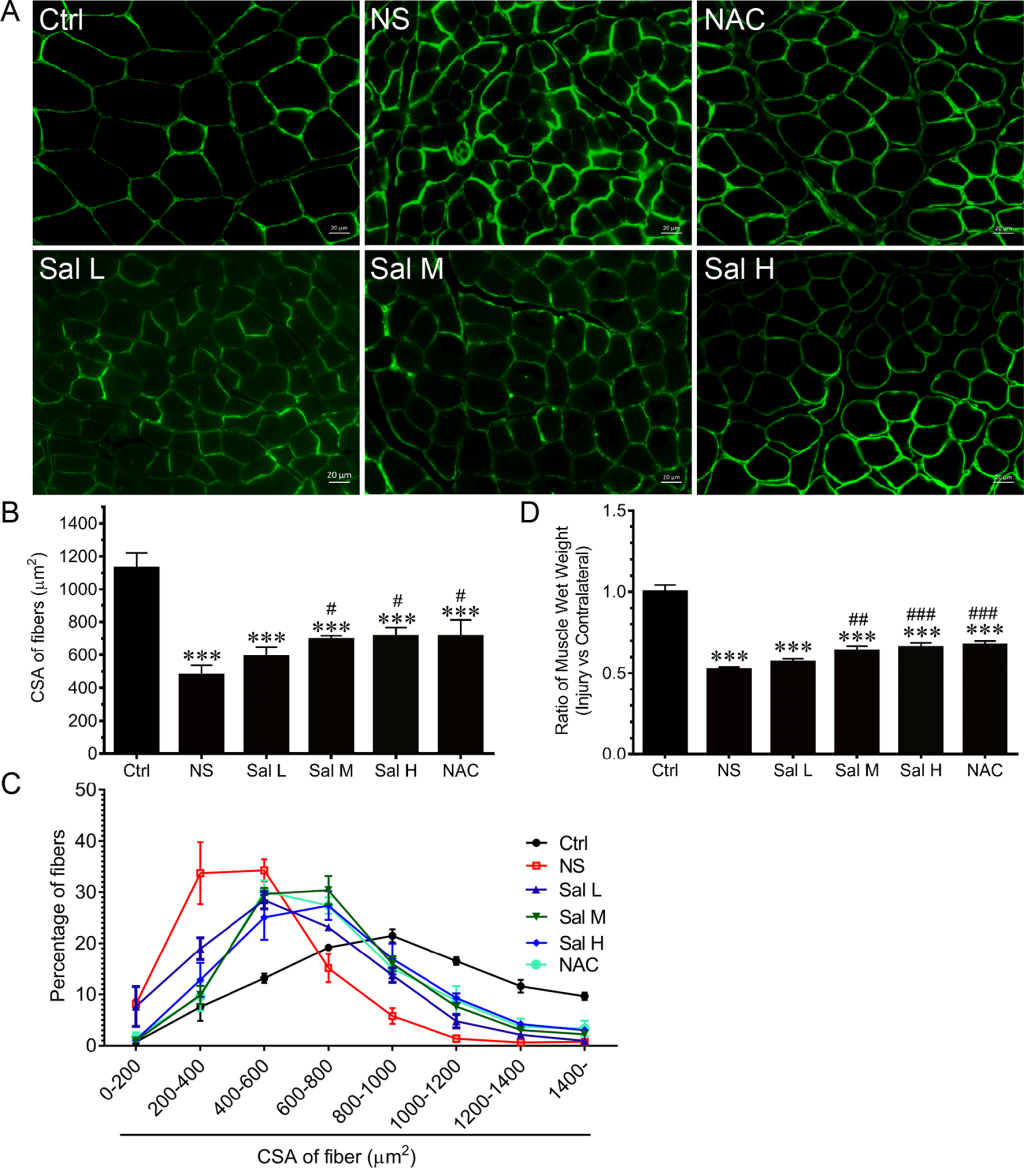
Salidroside alleviated denervation-induced skeletal muscle atrophy. After denervation, mice were injected intraperitoneally with saline vehicle plus salidroside (Sal L: 5 mg/kg/day, Sal M: 10 mg/kg/day, Sal H: 20 mg/kg/day), saline vehicle plus NAC (20 mg/kg/day; NAC), or saline vehicle only (NS) for 14 days. After sham operation, mice were injected intraperitoneally with saline vehicle (Ctrl) for 14 days. Then the tibialis anterior (TA) muscles were harvested to undergo laminin staining analysis. **(A)** Representative images of laminin-stained TA muscle cross-sections. Green indicates laminin staining. Scale bar, 20 μm. **(B)** Mean ± SD of CSA in TA fibers from each group. **(C)** Frequency distribution of CSA of TA muscles. **(D)** The ratio of TA muscles wet weight. Data are expressed as mean ± SD. ***p < 0.001 versus Ctrl. ^#^p < 0.05, ^##^p < 0.01, and ^###^p < 0.001 versus NS. n = 6 mice in each group.

To examine whether salidroside protects against denervation-induced skeletal muscle atrophy, we determined the mass and CSA of TA muscles from denervated mice treated with or without salidroside (Sal L: 5 mg/kg/day, Sal M: 10 mg/kg/day, Sal H: 20 mg/kg/day). After 14 days of salidroside administration, the denervation-induced loss of muscle mass was significantly blocked in the mice administrated with salidroside, which was confirmed by the increased TA muscle mass and CSA in the mice treated with salidroside, and the frequency distribution of CSA of fibers in TA muscles shifted toward bigger sizes. Moreover, the high doses of salidroside showed better performance in attenuating denervation-induced skeletal muscle atrophy (**Figure 4**). Therefore, high doses of salidroside were chosen in subsequent *in vivo* studies.

### Salidroside Inhibits Oxidative Stress and Inflammation in Denervated Muscles

**Figure 5:**
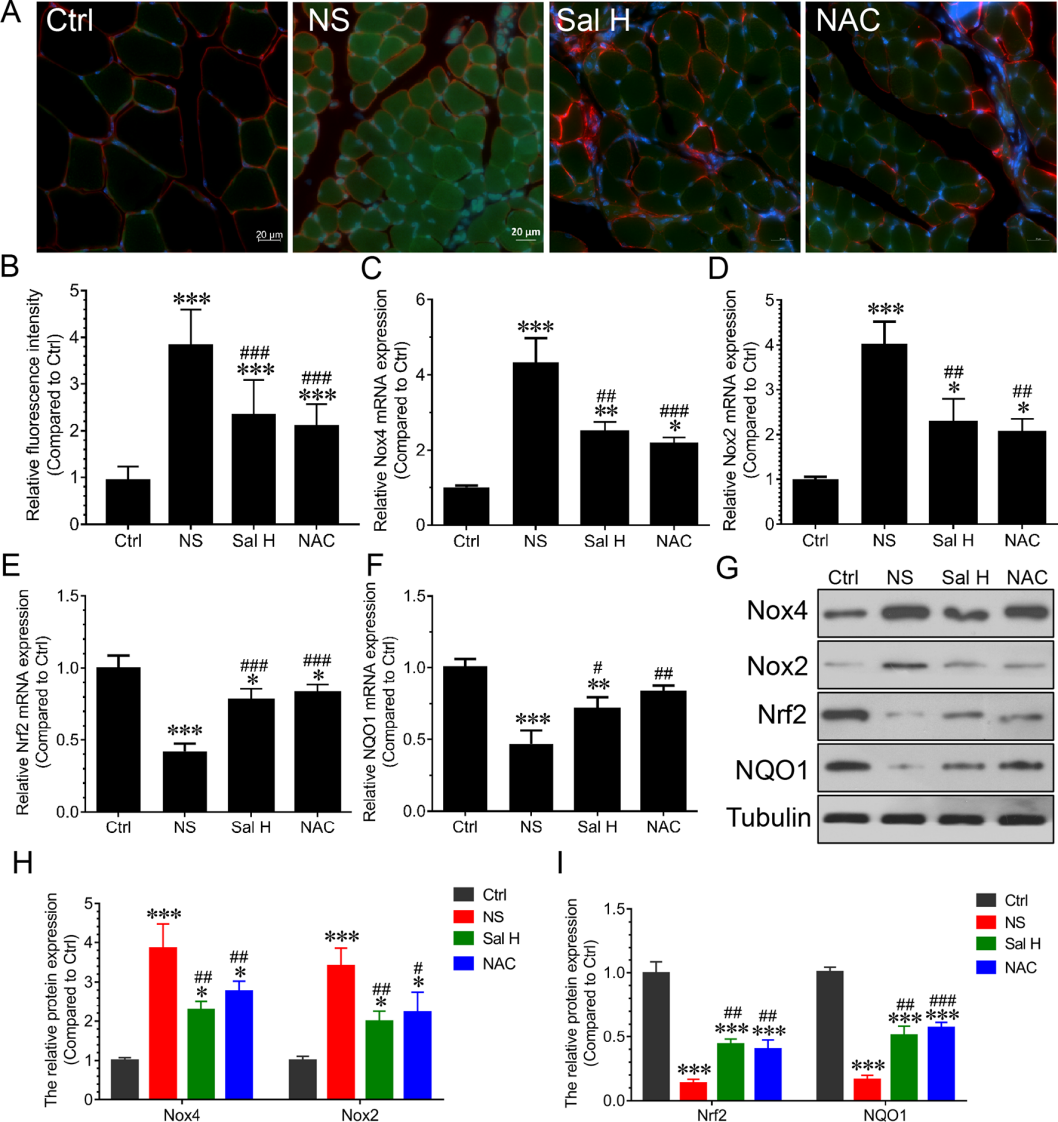
Salidroside relieved oxidative stress in skeletal muscles suffered from sciatic nerve transection. After denervation, mice were injected intraperitoneally with saline vehicle plus salidroside (Sal H: 20 mg/kg/day), saline vehicle plus NAC (20 mg/kg/day; NAC), or saline vehicle only (NS) for 14 days. After sham operation, mice were injected with saline vehicle (Ctrl) for 14 days. Then, the TA muscles were harvested to undergo DCF staining analysis. **(A)** Representative images of DCF-stained TA muscles cross-sections. Green and red indicate DCF staining and laminin staining, respectively. Scale bar, 20 μm. **(B)** Quantification of relative DCF fluorescence intensity. **(C**–**F)** The quantitative real-time polymerase chain reaction (qRT-PCR) analyses of the expression of oxidative stress-related genes Nox2, Nox4, Nrf2, and NQO1 mRNA in TA muscles. **(G)** The expression of Nox2, Nox4, Nrf2, and NQO1 in TA muscles was determined by Western blotting. **(H**, **I)** Protein levels of Nox2, Nox4, Nrf2, and NQO1 in TA muscles. Data are expressed as mean ± SD. *p < 0.05, **p < 0.01, and ***p < 0.001 versus Ctrl. ^#^p < 0.05, ^##^p < 0.01, and ^###^p < 0.001 versus NS.

Previous studies revealed that oxidative stress and inflammation contributed to activation of Foxo3A during muscle atrophy (Wang et al., 2014; Kuczmarski et al., 2018; Lawler et al., 2019). To evaluate whether salidroside inhibits oxidative stress and inflammation in denervated muscles, we examined ROS levels and inflammatory cytokines. Our data showed that ROS were markedly induced in the denervated TA muscles, as evidenced by the increased DCF fluorescence. Interestingly, salidroside could inhibit the production of ROS in the denervated TA muscles, which was confirmed by the decreased DCF fluorescence (**Figures 5A, B**). At the same time, Nox2, Nox4, Nrf2, and NQO1were determined, and the results indicated that Nox2 and Nox4 mRNA and proteins were markedly induced, and Nrf2 and NQO1 mRNA and proteins were markedly reduced in the denervated TA muscles. Interestingly, salidroside could reverse the expression of Nox2, Nox4, Nrf2, and NQO1 in the denervated TA muscles (**Figures 5C–I**). Furthermore, the effects of salidroside on oxidative stress in denervated TA muscles were also observed in the denervated TA muscles treated with NAC (**Figure 5**).

**Figure 6:**
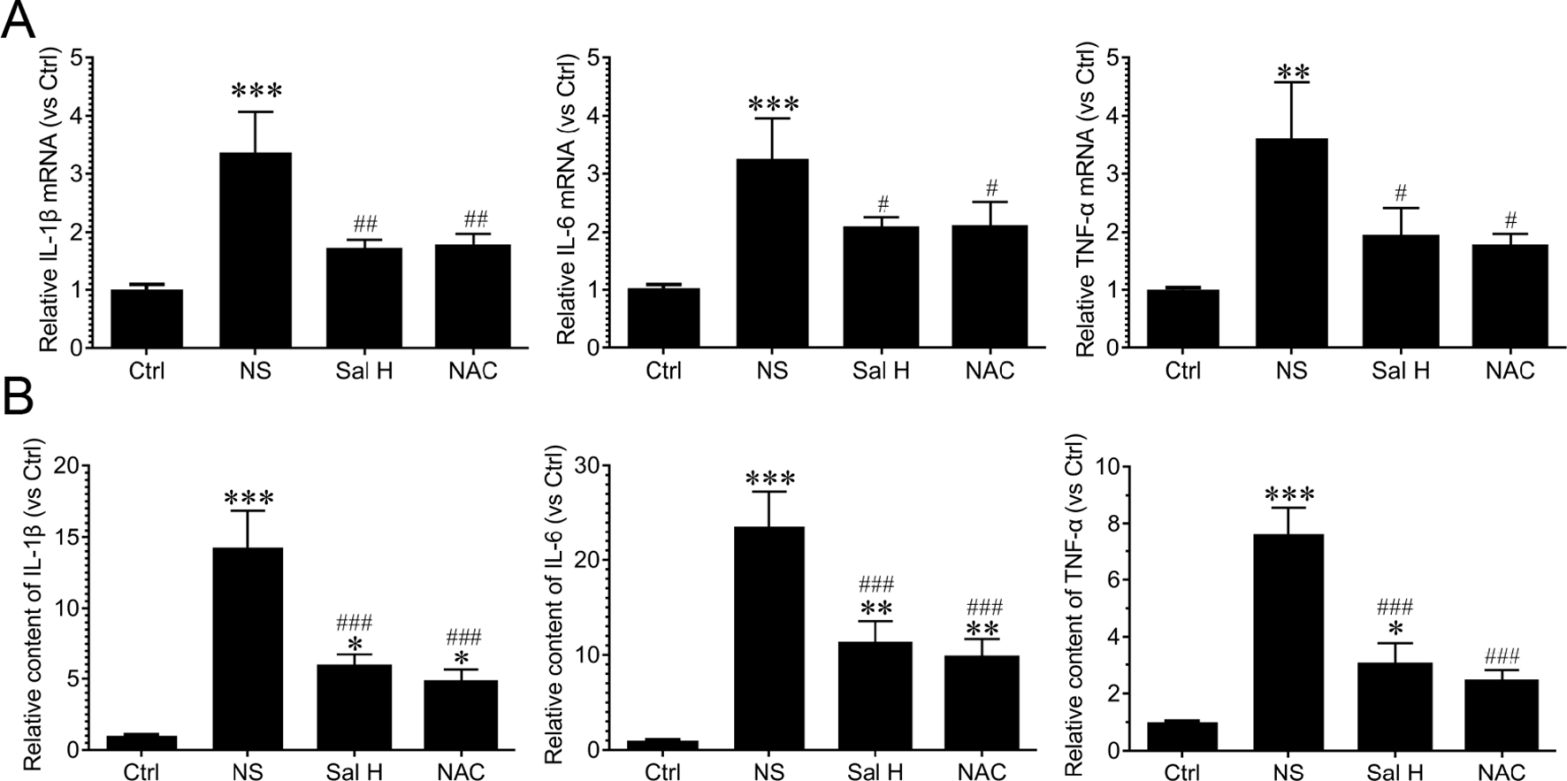
Salidroside suppressed inflammatory cytokines in skeletal muscles suffered from sciatic nerve transection. After denervation, mice were injected intraperitoneally with saline vehicle plus salidroside (Sal H: 20 mg/kg/day), saline vehicle plus NAC (20 mg/kg/day; NAC), or saline vehicle only (NS) for 14 days. After sham operation, mice were injected intraperitoneally with saline vehicle (Ctrl) for 14 days. Then, the inflammatory cytokines were determined through ELISA and qRT-PCR analyses. **(A)** The qRT-PCR analyses of the expression of inflammatory cytokines IL-1β, IL-6, and TNF-α in TA muscles. **(B)** The content of inflammatory cytokines, including IL-1β, IL-6, and TNF-α, were determined by ELISA. Data are expressed as mean ± SD. *p < 0.05, **p < 0.01, and ***p < 0.001 versus Ctrl. ^#^p < 0.05, ^##^p < 0.01, and ^###^p < 0.001 versus NS.

Our data also demonstrated that inflammation was significantly induced during denervation-induced muscle atrophy, as evidenced by the increased levels of inflammatory cytokines, such as IL-1β, IL-6, and TNF-α. Interestingly, salidroside suppressed the production of inflammatory cytokines. Moreover, the responses from salidroside inhibiting inflammatory cytokines were similar to the responses from NAC in the denervated TA muscles (**Figure 6**). These data suggest that salidroside-attenuated muscle atrophy is mediated, at least in part, through inhibiting oxidative stress and inflammation in denervated muscles.

### Salidroside Suppresses Foxo3A in Denervation-Induced Skeletal Muscle Atrophy

**Figure 7:**
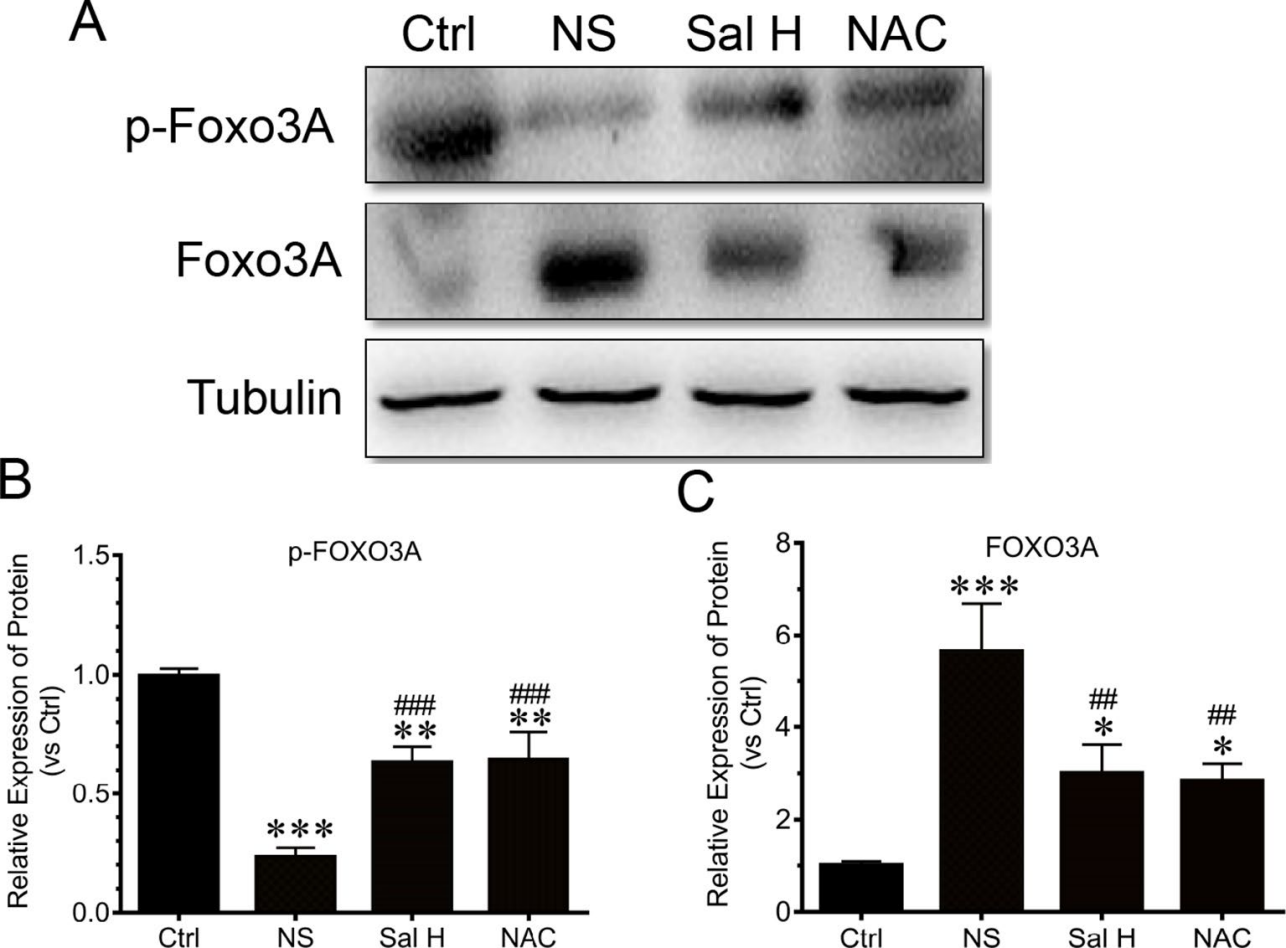
Salidroside improved the expression of transcripitional factor forkhead box O3 A (Foxo3A) and phosphorylated Foxo3A in TA muscles of mice suffered from sciatic nerve transection. After denervation, mice were injected intraperitoneally with saline vehicle plus salidroside (Sal H: 20 mg/kg/day), saline vehicle plus NAC (20 mg/kg/day; NAC), or saline vehicle only (NS) for 14 days. After sham operation, mice were injected intraperitoneally with saline vehicle (Ctrl) for 14 days. Then the TA muscles were harvested to undergo Western blotting analysis. **(A)** Representative Western blot images were shown for Foxo3A and phosphorylated Foxo3A. **(B)** Histograms comparing phosphorylated Foxo3A levels among different muscle samples. **(C)** Histograms comparing Foxo3A levels among different muscle samples. Data are expressed as mean ± SD. *p < 0.05, **p < 0.01, and ***p < 0.001 versus Ctrl. ^##^p < 0.01 and ^###^p < 0.001 versus NS.

Foxo3A has been implicated as a major mediator of muscle atrophy through activation of the ubiquitin–proteasome pathway (Wei et al., 2013). The phosphorylation of Foxo3A leads to the blockade of the upregulation of MuRF1 and MAFbx (Sakai et al., 2014). To understand whether salidroside regulates Foxo3A transcription factors, we determined the expression of Foxo3A and phosphorylated Foxo3A. Our data demonstrated that Foxo3A displayed a significant increase in the TA muscles of denervated mice, while the phosphorylated Foxo3A displayed a significant decrease (**Figure 7**). Interestingly, salidroside could reverse the increase of Foxo3A and the reduction of phosphorylated Foxo3A in TA muscles of denervated mice. Moreover, these effects of salidroside on the expression of Foxo3A and phosphorylated Foxo3A in denervated TA muscles were also observed in the denervated TA muscles treated with NAC (**Figure 7**). In summary, we might speculate that salidroside suppresses Foxo3A and enhances the phosphorylation of Foxo3A in denervated TA muscles. These results suggested that salidroside-attenuated proteolysis is mediated, at least in part, through regulation of the expression of Foxo3A and phosphorylated Foxo3A in denervated TA muscles.

### Salidroside Inhibits Ubiquitin–Proteasome System in Denervation-Induced Skeletal Muscle Atrophy

**Figure 8:**
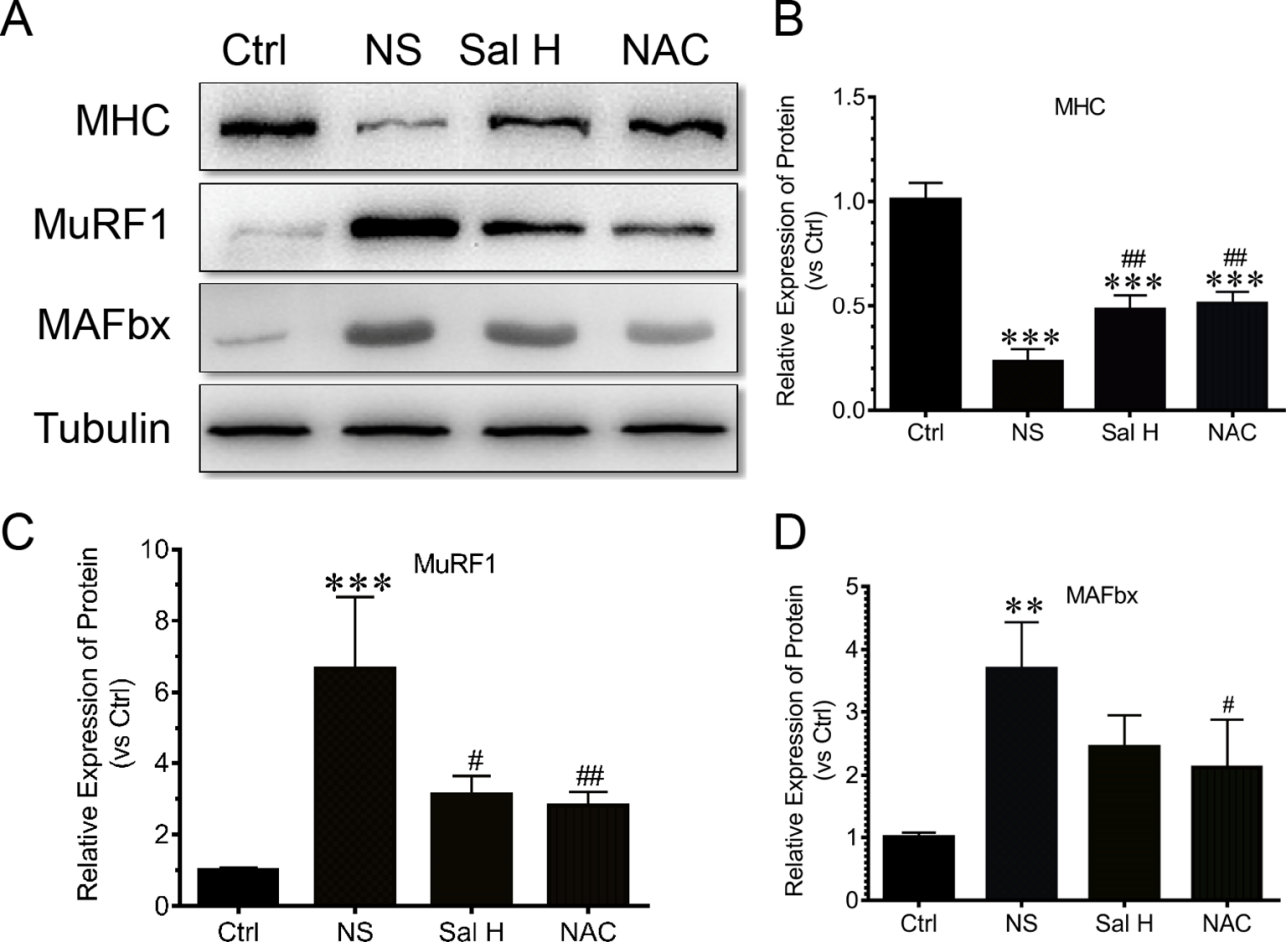
Salidroside inhibited the expression of ubiquitin–proteasome pathway—especially E3 ubiquitin ligases muscle-specific F-box (MAFbx) and muscle ring-finger protein-1 (MuRF1), and improved the expression of MHC in TA muscles of mice suffered from sciatic nerve transection. **(A)** Representative blots of MHC, MAFbx, and MuRF1 in TA muscles. **(B)** Relative expression of MHC in TA muscles. **(C)** Relative expression of MuRF1 in TA muscles. **(D)** Relative expression of MAFbx in TA muscles. Data are expressed as mean ± SD. **p < 0.01 and ***p < 0.001 versus Ctrl. ^#^p < 0.05 and ^##^p < 0.01 versus NS.

To assess how salidroside attenuates denervation-induced muscle atrophy, we examined evidences for the ubiquitin–proteasome system proteolytic and protein synthesis activities. Interestingly, the elevated expression of muscle-specific E3 ubiquitin ligases Atrogin-1/MAFbx and MuRF1 was suppressed by salidroside in denervated muscles (**Figure 8**). Intriguingly, these responses from salidroside were also observed in the muscles of NAC administration (**Figure 8**). These data suggested that salidroside attenuated denervation-induced skeletal muscle atrophy through inhibition of the ubiquitin–proteasome proteolytic pathway.

### Salidroside Inhibits Mitophagy in Denervated Skeletal Muscle

**Figure 9:**
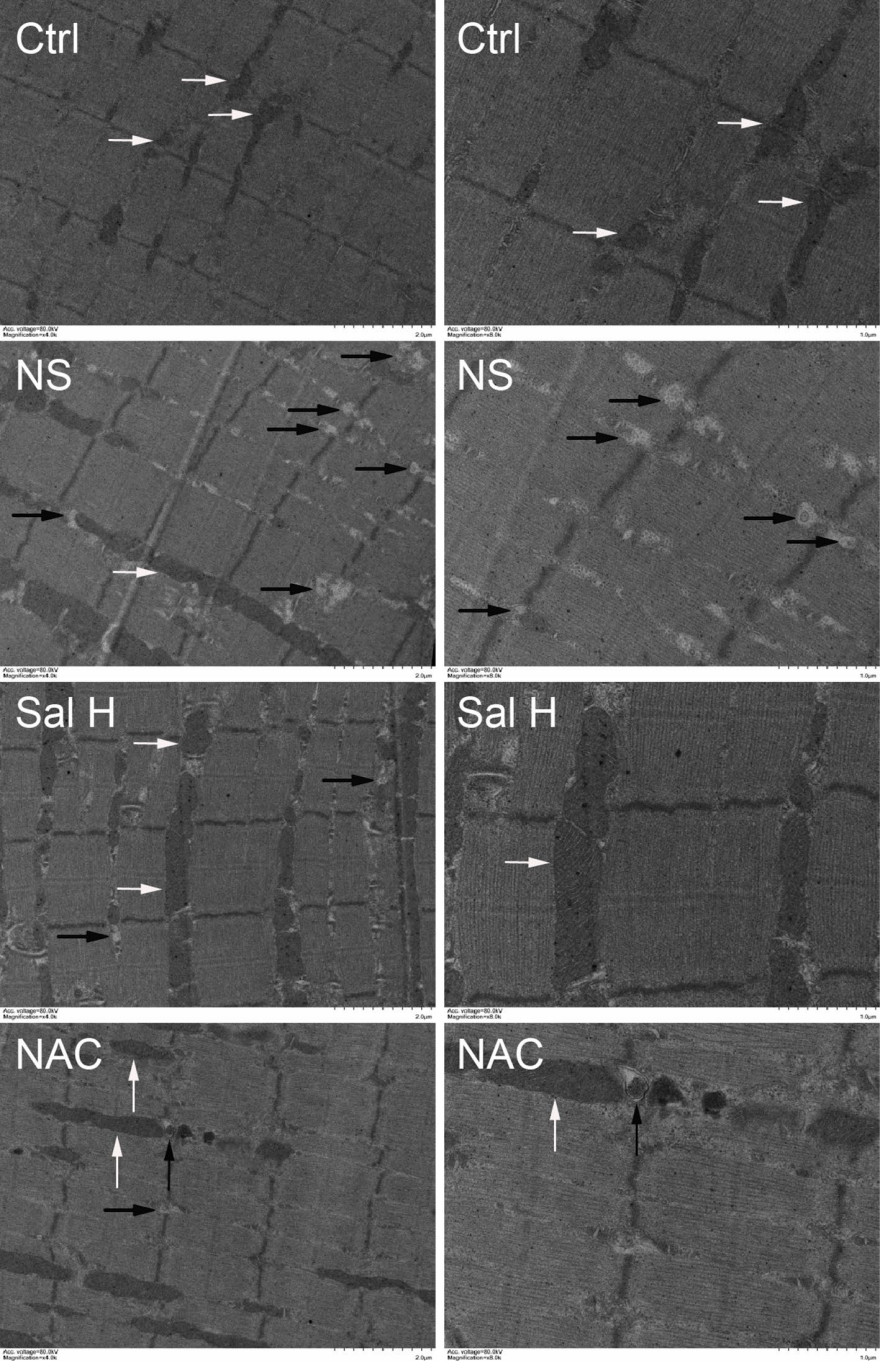
Salidroside inhibited mitophagy in the TA muscles following sciatic nerve transection. After denervation, mice were injected intraperitoneally with saline vehicle plus salidroside (Sal H: 20 mg/kg/day), saline vehicle plus NAC (20 mg/kg/day; NAC), or saline vehicle only (NS) for 14 days. After sham operation, mice were injected intraperitoneally with saline vehicle (Ctrl) for 14 days. Then the TA muscles were harvested to undergo transmission electron microscopy (TEM) analysis. Representative TEM micrographs of TA muscles. The right is a local enlargement of the left. White arrows indicate normal mitochondria. Black arrows indicate mitophagy.

**Figure 10:**
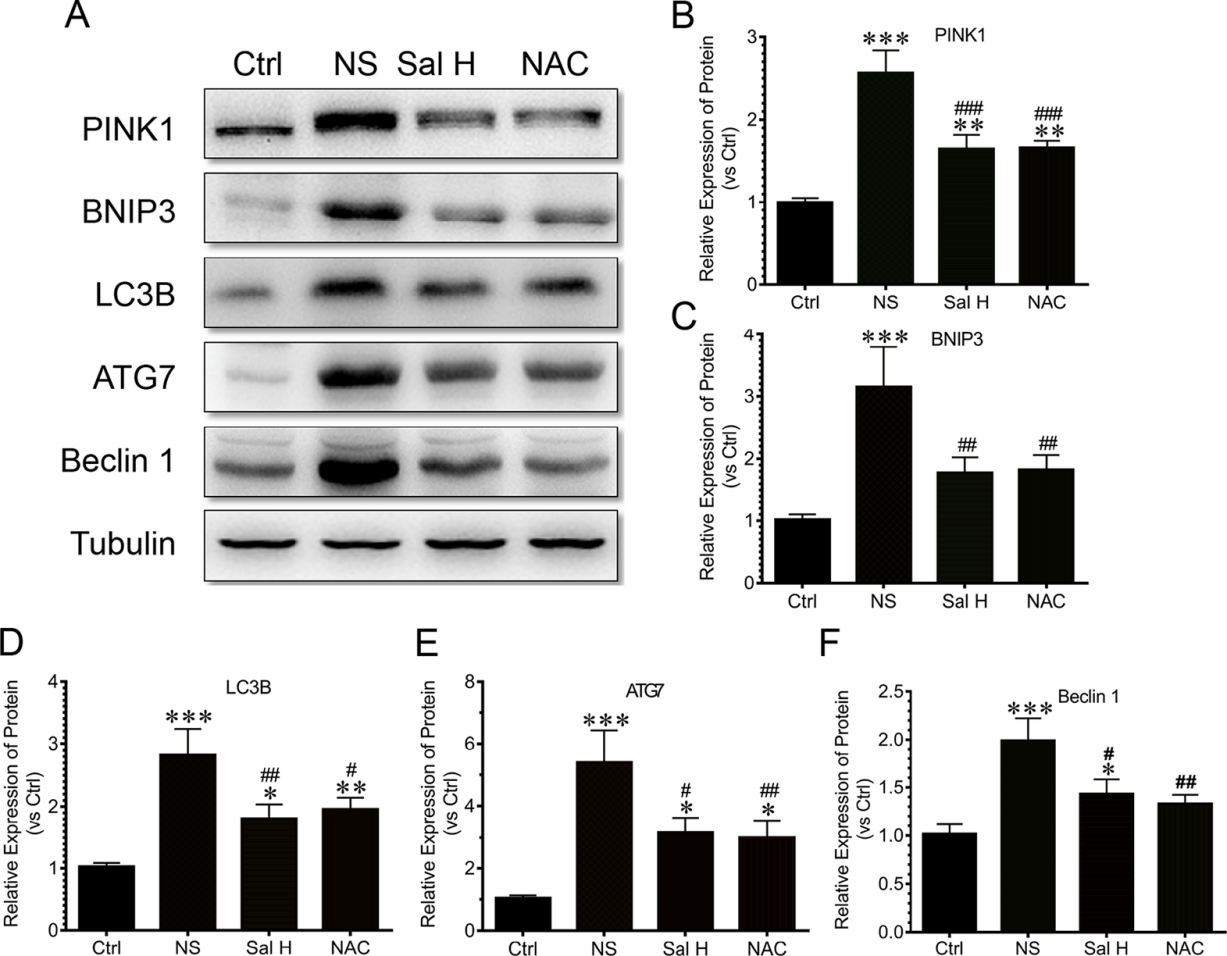
Salidroside inhibited the expression of autophagy genes, including PTEN-induced putative kinase 1 (PINK1), Bcl2/adenovirus E1B 19-kDa protein-interacting protein 3 (BNIP3), microtubule-associated protein 1 light chain 3 beta (LC3B), autophagy-related protein 7 (ATG7), and Beclin1, in TA muscles of mice suffered from sciatic nerve transection. **(A)** Representative blots of PINK1, BNIP3, LC3B, ATG7, and Beclin1 in TA muscles. **(B)** Relative expression of PINK1 in TA muscles. **(C)** Relative expression of BNIP3 in TA muscles. **(D)** Relative expression of LC3B in TA muscles. **(E)** Relative expression of ATG7 in TA muscles. **(F)** Relative expression of Beclin1 in TA muscles. Data are expressed as mean ± SD. *p < 0.05, **p < 0.01, and ***p < 0.001 versus Ctrl. ^#^p < 0.05, ^##^p < 0.01, and ^###^p < 0.001 versus NS.

To access whether salidroside inhibits mitophagy during denervation-induced skeletal muscle atrophy, we studied the effects of salidroside on autophages or autophagic vesicles and mitophagy markers (i.e., PINK1, BNIP3, LC3B, ATG7, and Beclin1) in denervated muscles. Mitophagy was significantly induced in denervated skeletal muscles, as evidenced by increased autophages or autophagic vesicles and elevated mitophagy markers, including PINK1, BNIP3, LC3B, ATG7, and Beclin1. Interestingly, salidroside inhibited the production of autophages or autophagic vesicles, accompanied by the decreased mitophagy markers. The responses from salidroside on mitophagy in denervated TA muscles were also observed in the denervated TA muscles treated with NAC (**Figures 9** and **10**). These data indicated that salidroside-inhibited mitophagy through the decreased expression of mitophagy markers.

## Discussion

**Figure 11:**
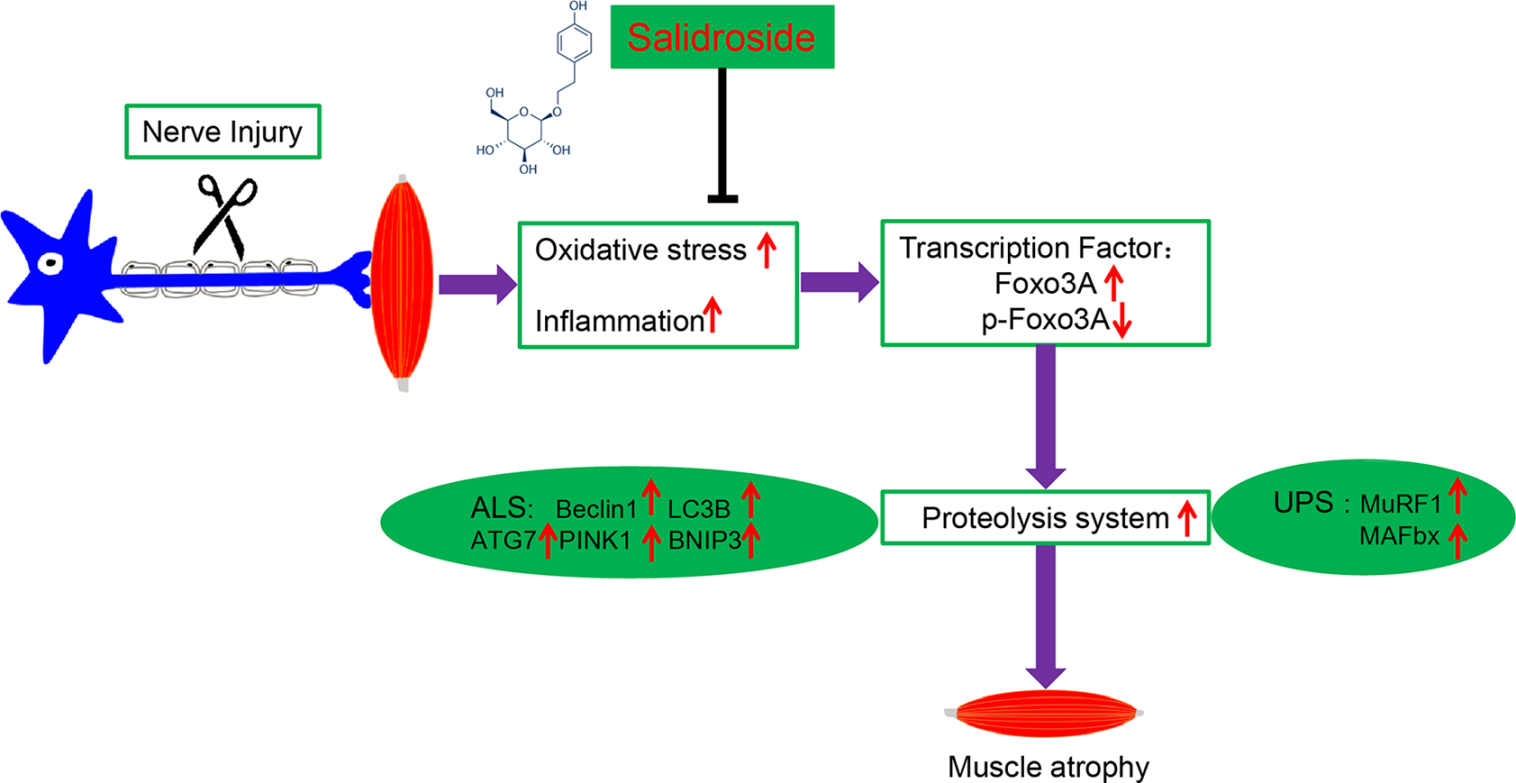
A schematic diagram illustrating the proposed mechanism by which salidroside attenuates denervation-induced skeletal muscle atrophy. Denervation-induced skeletal muscle atrophy is associated with elevated oxidative stress and inflammation, elevated Foxo3A, and decreased p-Foxo3A, greater activation of proteolysis and mitophagy in this study. Interestingly, salidroside improved skeletal muscle atrophy by inhibiting oxidative stress, inflammation and mitophagy, and giving rise to reduced proteolysis in denervated skeletal muscle.

In the present study, we demonstrated for the first time that salidroside administration attenuated muscle atrophy and inhibited mitophagy by suppressing the activation of the UPS and ALS in the TA muscles from denervated mice. Meanwhile, we demonstrated that salidroside administration reduced the expression of Foxo3A, a major mediator of muscle atrophy, by suppressing oxidative stress and inflammation in the skeletal muscle of denervated mice (**Figure 11**).

Appropriate levels of ROS assist in mounting an effective defense against pathogens. However, sustained overproduction of ROS is believed to conduce to cellular damage (Chen et al., 2017). Overproduction of ROS and pro-inflammatory cytokines are two main molecular mechanisms involved in muscle atrophy (van Helvoort et al., 2007; Kim et al., 2015; Anderson et al., 2017; Ma et al., 2018), which suggests that anti-inflammation or antioxidation may be an effective treatment for muscular atrophy. In the current study, we showed that nutrition deprivation markedly induced ROS and inflammatory response in myotubes, as evidenced by elevated ROS content and increased inflammatory cytokines. These results demonstrated that ROS and inflammation played a crucial role in nutrition deprivation-induced myotube atrophy. Salidroside is a principal active ingredient of *R. rosea* and has been reported to have cellular protection through antioxidation and the inhibition of free radicals (Mao et al., 2015). Being free of side effects makes salidroside potentially attractive as a candidate drug for the treatment of many disorders. Chen et al. demonstrated that salidroside could suppress LPS-induced myocardial injury through inhibition of ROS and inflammation (Chen et al., 2017). Interestingly, our data showed that salidroside alleviated myotube atrophy induced by nutrition deprivation, which was confirmed by the increased myotube diameter. Meanwhile, the ROS content and the levels of inflammatory cytokines were reduced by the treatment with salidroside. Furthermore, ROS scavenger NAC obtained similar effects with salidroside in the myotubes suffered from nutrition deprivation, which suggested that salidroside could protect against nutrition deprivation-induced myotube atrophy, at least in part, through inhibiting oxidative stress and inflammation. This phenomenon makes us want to know whether salidroside also inhibits oxidative stress and inflammation during denervation-induced muscle atrophy, thereby protecting against muscle atrophy.

So we want to know whether salidroside could inhibit muscle atrophy by inhibiting ROS and inflammation. In the current study, we found that salidroside blocked the denervation-induced loss of muscle mass. Moreover, the high doses of salidroside showed better performance in attenuating muscle atrophy. This study also showed that ROS and inflammation were markedly induced in the denervated TA muscles, which suggested that ROS and inflammation might play a crucial role in denervation-induced muscle atrophy. This phenomenon also occurs in muscular atrophy caused by other factors, such as chronic kidney disease and aging (Enoki et al., 2016; Zhao et al., 2018). Interestingly, our study showed that salidroside could inhibit the production of ROS and inflammatory cytokines in the denervated TA muscles. The antioxidant and anti-inflammatory properties of salidroside were consistent with previous studies, which demonstrated that salidroside protected against Parkinson’s disease and Alzheimer’s disease, and promoted random skin flap survival through inhibiting oxidative stress and inflammation (Zhang et al., 2013; Deheng et al., 2016; Zhou et al., 2019). Moreover, the responses from salidroside inhibiting oxidative stress and inflammation were similar to the responses from NAC in the denervated TA muscles, which also suggested that inflammation might be downstream of oxidative stress. These data suggested that salidroside could inhibit oxidative stress and inflammation in the skeletal muscles suffered from denervation. Additionally, previous studies revealed that oxidative stress and inflammation contributed to activation of Foxo3A during muscle atrophy (Wang et al., 2014; Kuczmarski et al., 2018; Lawler et al., 2019). Therefore, we want to explore whether salidroside can inhibit the activation of Foxo3A in denervation-induced muscle atrophy.

Foxo3A has been implicated as a major mediator of muscle atrophy through activation of the ubiquitin–proteasome pathway (Wei et al., 2013). The current study demonstrated that Foxo3A displayed a significant increase in the TA muscles of denervated mice, while the phosphorylated Foxo3A displayed a significant decrease, which was consistent with other study. It showed that the expression of Foxo3A was increased, while p-Foxo3A expression decreased gradually in TA muscles after denervation (Sei et al., 2015). Interestingly, salidroside reversed the increase in Foxo3A and the reduction in p-Foxo3A in denervated TA muscles. Moreover, these effects of salidroside on the expression of Foxo3A and p-Foxo3A were similar to that observed in the denervated TA muscles treated with NAC. These results suggested that salidroside inhibit the activation of Foxo3A, at least in part, through inhibition of ROS and inflammation. The activation of Foxo was essential for the muscle atrophy induced by denervation or fasting, and activated Foxo3 caused coordinately activating UPS and ALS (Zhao et al., 2007; Zhao et al., 2008). Therefore, we want to know whether salidroside could protect against denervated muscle atrophy by inhibiting UPS and ALS.

In the current study, we found that salidroside blocked the denervation-induced loss of muscle mass. Moreover, the high doses of salidroside showed better performance in attenuating muscle atrophy. Previous studies demonstrated that skeletal muscle atrophy was accompanied by the activation of proteolytic pathways UPS and ALS, as evidenced by elevated levels of key enzymes of UPS and key effectors of ALS (Paul et al., 2010; Steiner et al., 2015; Ceelen et al., 2017; Ito et al., 2017). In the present study, we found that the elevated expression of MAFbx and MuRF1 was suppressed and reversed by salidroside in denervated muscles, which indicated that salidroside suppressed the ubiquitin–proteasome proteolytic pathway. Intriguingly, these responses from salidroside were also observed in the muscles of NAC administration, which suggested that salidroside might play its role by inhibiting ROS.

Mitophagy is critical for skeletal muscle function, and impaired mitophagy leads to an accumulation of damaged mitochondria, which can negatively regulate muscle mass (Wang et al., 2018). Our data showed that mitophagy was significantly induced in denervated skeletal muscles, and salidroside inhibited the production of autophages or autophagic vesicles, accompanied by the decreased mitophagy markers. These results were consistent with other studies, which demonstrated that salidroside protected cortical neurons against glutamate-induced cytotoxicity by inhibiting autophagy (Yin et al., 2016), and salidroside pretreatment attenuated autophagy during hepatic ischemia–reperfusion injury (Feng et al., 2017). Nevertheless, the effects of salidroside suppressing mitophagy were simulated by NAC in the denervated TA muscles, which also suggested that salidroside alleviated mitophagy by inhibiting ROS.

In summary, we conclude that denervation induces oxidative stress and inflammation, which elevates the expression of Foxo3A and decreases the expression of p-Foxo3A, contributing to the activation of proteolytic pathways. We, for the first time, demonstrate that salidroside alleviates denervation-induced oxidative stress and inflammatory response, thereby inhibits muscle proteolysis, and finally attenuates denervation-induced muscle atrophy (**Figure 11**). Antioxidation and anti-inflammation may be effective therapeutic targets for denervated muscle atrophy. Salidroside may be a potential therapeutic candidate to prevent muscle wasting. These results uncover a new application for salidroside.

## Data Availability

The datasets generated for this study are available on request to the corresponding author.

## Author Contributions

HS designed the study. ZH, QF, WM, QZ, and JQ performed the experiments. ZH, QF, WM, and QZ collected and assembled the data. ZH, QF, and WM performed data analysis. XG and HY provided scientific expertise. HS and ZH wrote the manuscript.

## Funding

This work was supported by the National Key Research and Development Program of China (Grant No. 2017YFA0104703), National Natural Science Foundation of China (Grant Nos. 81871554, 81671230, 31730031), the 973 Program (Grant Nos. 2014CB542202 and 2014CB542203), a project funded by Jiangsu Provincial Key Medical Center, and the Priority Academic Program Development of Jiangsu Higher Education Institutions (PAPD).

## Conflict of Interest Statement

The authors declare that the research was conducted in the absence of any commercial or financial relationships that could be construed as a potential conflict of interest.

## Abbreviations

MuRF1, muscle ring-finger protein-1; MAFbx, muscle-specific F-box; MHC, myosin heavy chain; TA, tibialis anterior; mTOR, mammalian target of rapamycin; mTORC1, mammalian target of rapamycin complex 1; ROS, reactive oxygen species; BNIP3, Bcl2/adenovirus E1B 19-kDa protein-interacting protein 3; PINK1, PTEN-induced putative kinase 1; Foxo3A, forkhead box O3 A; LC3B, microtubule-associated protein 1 light chain 3 beta; ATG7, autophagy-related protein 7; UPS, ubiquitin–proteasome system; ALS, autophagy–lysosomal system; TNF-α, tumor necrosis factor alpha; IL6, interleukin-6; IL1β, interleukin 1 beta; Nox2, NADPH oxidase 2; Nox4, NADPH oxidase 4; Nrf2, nuclear factor (erythroid-derived 2)-like 2; NQO1, NAD(P)H dehydrogenase [quinone] 1; HO-1, Heme oxygenase 1; HBSS, Hank’s balanced salt solution; DHE, dihydroethidium; DCFH-DA,dichlorodihydrofluorescein diacetate; NAC, N-acetyl-cysteine; SD, standard deviation; ELISA, enzyme-linked immunosorbent assay; TEM, transmission electron microscopy; qRT-PCR, quantitative real-time polymerase chain reaction; RIPA, radio immunoprecipitation assay; SDS-PAGE, sodium dodecyl sulfate-polyacrylamide gel electrophoresis; DMEM, Dulbecco’s modified Eagle’s medium; FBS, fetal bovine serum; FoxO, forkhead box class O; PBS, phosphate-buffered saline.
